# Deep convolutional neural networks using an active learning strategy for cervical cancer screening and diagnosis 

**DOI:** 10.3389/fbinf.2023.1101667

**Published:** 2023-03-09

**Authors:** Xueguang Li, Mingyue Du, Shanru Zuo, Mingqing Zhou, Qiyao Peng, Ziyao Chen, Junhua Zhou, Quanyuan He

**Affiliations:** The Key Laboratory of Model Animals and Stem Cell Biology in Hunan Province, School of Medicine, Hunan Normal University, Changsha, Hunan, China

**Keywords:** cervical cancer, CNN, active learning strategy, deep learning, whole slide image

## Abstract

Cervical cancer (CC) is the fourth most common malignant tumor among women worldwide. Constructing a high-accuracy deep convolutional neural network (DCNN) for cervical cancer screening and diagnosis is important for the successful prevention of cervical cancer. In this work, we proposed a robust DCNN for cervical cancer screening using whole-slide images (WSI) of ThinPrep cytologic test (TCT) slides from 211 cervical cancer and 189 normal patients. We used an active learning strategy to improve the efficiency and accuracy of image labeling. The sensitivity, specificity, and accuracy of the best model were 96.21%, 98.95%, and 97.5% for CC patient identification respectively. Our results also demonstrated that the active learning strategy was superior to the traditional supervised learning strategy in cost reduction and improvement of image labeling quality. The related data and source code are freely available at https://github.com/hqyone/cancer_rcnn.

## 1 Background

Cervical cancer (CC) is the fourth most common malignant tumor among women worldwide, with an estimated 0.53 million new cases and 0.27 million deaths each year. The ThinPrep cytologic test (TCT) was introduced in the 1990s to screen for the presence of atypical cells, cervical cancer, or precursor lesions (LSIL, HSIL) as well as other cytologic categories as defined by the Bethesda System (Pangarkar, 2022) for routine screening and diagnosis of cervical cancer. The test requires trained pathologists to microscopically check for changes in abnormal squamous cells in the cytoplasm, nuclear shape, and fluid base color. The misdiagnosis or missed diagnosis in traditional manual slide reading of cervical cytology may occur to different extents due to differences in pathologist experience and technical level, or due to other factors such as fatigue,. The false-negative rate of manual interpretation is as high as 10%, the sensitivity of precancerous lesions detection is only about 65%, and the specificity is about 90%.

Deep convolutional neural networks, as a revolutionary technology, have been widely and successfully applied in medical data analysis and computer-aided diagnosis (CAD) (Shen et al., 2017). Recently, the combination of deep learning models with whole-slide images (WSI) scanning technologies has enabled automatic and remote disease diagnosis, reduced labor costs, and improved diagnostic accuracy. Deep learning technology has also been used in reading TCT slides from patients with cervical cancer, with promising results in reducing pathologist labor and improving diagnostic accuracy (Wu et al., 2018; Sompawong et al., 2019; Tan et al., 2021). Although these models have been reported to achieve high sensitivity and specificity, the generalization of these DCCN models requires further verification. Moreover, while a high diagnostic accuracy of >99% is absolutely required to avoid an omission of any patients, no deep learning models have yet achieved this level of accuracy (Esteva et al., 2017; Yoo et al., 2020; Lotter et al., 2021). Additionally, the models and training data of these studies usually are not publicly available, which makes the comparison and validation of these models difficult.

**FIGURE 1:**
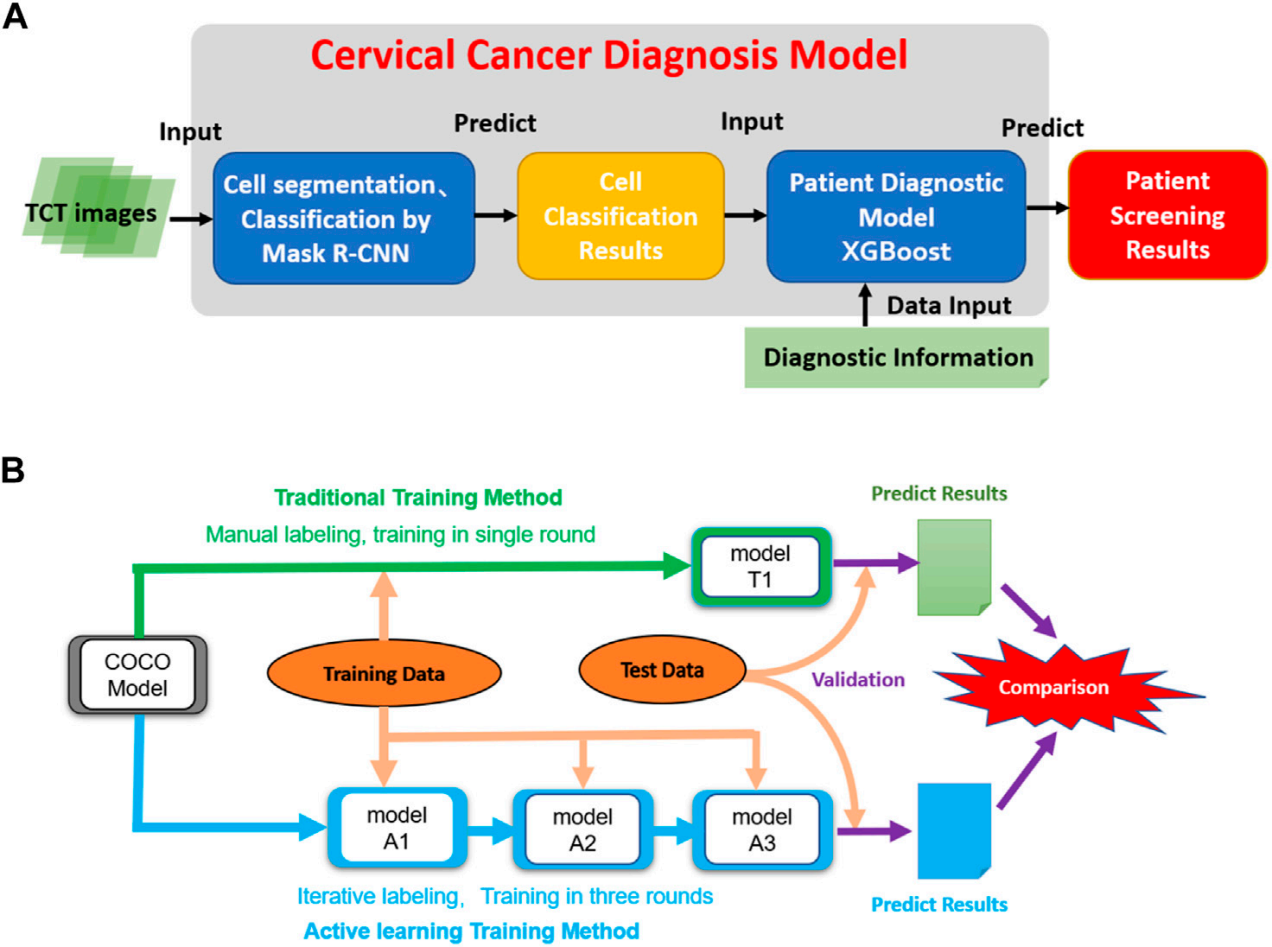
The architecture of the model and the dataflow of the project. **(A)** The architecture of the cervical cancer diagnosis model. It takes TCT images as inputs and then performs cell segmentation and classification. Then the cell classification results combined with related clinic diagnostic information are used to do patient classification. **(B)** The data process workflow of this project. The initial COCO DCNN model was trained using traditional and active learning methods. Then two final cell segmentation/classification models (T1 and A3) were evaluated and compared to each other.

Usually, a large set of high-quality training images is required to construct a high-accuracy AI diagnostic model (Moen et al., 2019). The conventional supervised learning strategy includes two main stages: “image annotation” and “model training”. In the image annotation stage, many images must be labeled manually by high-level pathologists, which usually involves many repetitive tasks, which is time-consuming and of low efficiency. This barrier limits the scale and quality of training data and is the main bottleneck in the development of AI diagnosis models. Active learning is an iterative supervised learning method in which a learning algorithm can interactively query a user (pathologist) to label new data points with the desired outputs (Moen et al., 2019). Active learning is widely used in the scenario in which unlabeled data are abundant but manual labeling is expensive. Figure 1B shows an active learning model, in which a closed loop between “data labeling” and “model training” and the DCCN model is a key contributor to the interactive learning procedure to facilitate data labeling (Figure 1B), thus greatly reducing the time, improving the quality of data labeling, balancing the experience differences among experts, and improving the speed and accuracy of model training (Settles, 1995; Aggarwal et al., 2014).

The present study aimed to generate accurate DCCN models for cervical cancer diagnoses based on WSIs of TCT slides. We constructed a large WSI dataset from 211 patients with cervical cancer and 100 patients without cervical cancer. The results from reading pathologic TCT slides of patients with cervical cancer from independent sources showed the high diagnostic accuracy of our AI models (close to 100%), suggesting their suitability for clinical use. The comparison between conventional supervised and active learning strategies showed the significant advantages of the latter in the construction of labeled datasets in terms of the completeness and accuracy of image labeling and the accuracy of the AI model. These findings suggested that active learning strategies can be effectively extended to the construction of AI diagnostic models of other diseases.

## 2 Materials and methods

The study was reviewed and approved by the Biology and Medicine Research Ethics Committee (BMREC) of the Hunan Normal University (2022 (No. 266)). All experiments were performed in accordance with the relevant guidelines and regulations. With the informed consent of patients, the project collected the digital scan images of 400 cervical TCT slides from 400 patients in Hunan Province. Patient names and other personal information were anonymized and de-identified before analysis to protect patient privacy.

### 2.1 Experimental design

Our predictive model consists of two main phases: 1) using a Mask R-CNN model to segment and classify cells in all TCT images from patients into three classes (see above); and 2) using the number of cells in each type as features to classify patients into two classes (normal vs. abnormal). We first trained the Mask R-CNN model using the pre-labeled training images and then built a machine-learning model for patient classification using the combination of cell classification and clinic diagnosis results (Figure 1A).

In the cell classification phase, we compared the traditional and active training strategies in terms of the cost of model training and accuracy. A pre-trained Mask R-CNN model based on the MS COCO dataset was used as the initial model. The T1 model was trained using all labeling images in a single round. The active training procedures of the A1, A2, and A3 models included three iterations. First, the training image was split into three parts. In the first iteration, the MS COCO model was trained by 100 manual labeling images (model A1). Then, the 200 images labeled by model A1 and revised by the pathologists were used to train the A2 models based on model A1 in the second iteration. The third iteration used an additional 150 images and was run similarly to the second iteration to create model A3. We compared the active learning strategy models to the traditional labeling method using independent testing data (Figure 1B).

### 2.2 Image collection and selection

A total of 400 cervical TCT slides from 400 patients with final diagnosis results (211 CC and 189 non-CC patients) were collected at Ning Xiang People’s Hospital. The slides were stained following the standard Feulgen staining process (Bancroft, Gamble) and scanned using a digital pathology slide scanner (Pinsheng Biotechnology Co., Ltd.) to generate the WSIs. Each WSI was composed of 300 single-field images (micro magnification 400×; resolution: 1024 × 1024).

As the number of abnormal cells is much smaller than the number of normal cells and manual cell segmentation is very time-consuming, we manually selected 500 images containing at least one abnormal cell from patients with CC and 500 single-field images from normal patients (no abnormal cells) for downstream model training. All abnormal cells in the TCT images were diagnosed and labeled by at least two professional pathologists. Only images meeting the following criteria were included: clear field of vision, even cell distribution on the slide, and a moderate number of cells in the image with few overlapping cells.

### 2.3 Image augmentation

As the data samples in this study were limited, we used the python library “imgaug” 0.4.0 to perform the image augmentation to increase the data diversity and better simulate the real data variability while preventing overfitting (https://imgaug.readthedocs.io/en/latest/). During the data training procedure, for each training image, the image processing program randomly selected one of three methods (flip/mirror, PIL-like affine transformations, contrast changes) to apply to the original images to create a transformed image to double the number of images in the training process.

### 2.4 Cell classification

According to WHO diagnostic criteria and cytopathology standards (Haugen et al., 2016), all cells in the images were manually classified and labeled into three categories: 1) normal squamous epithelial cells with the following characteristics were labeled as “Yin”: nucleus medium in size, small nucleocytoplasmic ratio, and transparency; 2) ecological cells (suspected diseased cells) were labeled as “yin-yang”. The nucleocytoplasmic ratio of these cells was slightly larger than that of the normal cells. They had deep nuclear staining but neat nuclear edges and regular shape; 3) abnormal (diseased) cells were labeled as “yang”, and had a larger nucleocytoplasmic ratio than the ecological cells, dark and large nuclei, irregular karyotype, and unsmooth nuclear membranes. As most of the features of abnormal cells were derived from the nucleus and less overlapping between nuclei, we manually segmented and labeled the cell nucleus instead of the cytoplasm using the LabelMe software (Russell et al., 2008). The nucleus outlines and cell classification information were exported and stored in a JSON file for each image.

### 2.5 Cell nuclei segmentation and classification model

Mask R-CNN is an image segmentation algorithm based on a convolutional neural network (CNN) proposed by He et al. (2017). The model contains four steps: 1) first, the input image is extracted through the feature extraction network (Backbone) to obtain the feature map; 2) a predetermined number of ROIs (regions of interest) are then generated, which are based on each anchor in the feature map. Then, the ROIS are sent to a region proposal network (RPN) for binary classification (foreground or background) and bounding-box (BB) regression, which aims to fine-tune the target prediction box to make it closer to the real box. After removing the background ROIs according to the classification results; 3) ROI alignment is implemented for the remaining target ROIs. The ROIs were mapped accurately to the feature graph of the whole graph using the bilinear interpolation method. Finally; 4) classification (n-category classification), BB regression, and MASK generation were implemented using the fully connected network.

During training, the performance of the object detection was measured by the loss function, which was defined as follows (He et al., 2017):

$$L\left(p_{i},t_{i}\right)=\frac{1}{N_{cls}}\underset{i}{\sum }L_{cls}\left(p_{i},p_{i}^{*}\right)+\frac{\lambda }{N_{reg}}\underset{i}{\sum }p_{i}^{*}L_{reg}\left(t_{i},t_{i}^{*}\right)$$ (1)

where *i* is the subscript of an anchor in a mini-batch, $p_{i}$ is the prediction probability of an object in anchor *i*, $p_{i}^{*}$ is the ground truth label which is 1 if the anchor is positive and 0 otherwise. $t_{i}$ represents the coordinates of the bounding-box of the predicted target region; $t_{i}^{*}$ is the corresponding ground truth box’s parameterization coordinates. *L*_cls_ is the classification loss and *L*_reg_ represents regression loss. In our study, all cells in training data were divided into three categories (Ying, Ying-Yang, or Yang) based on the lesion degree. The algorithm encoded multiple categories as a continuous sequence (0,1,2). The classification loss is defined as follows:

$$L_{cls}\left(p_{i},p_{i}^{*}\right)=-\overset{N}{\underset{c}{\sum }}{\omega_{c}\times y}_{c}\times \log\left(p_{c}\right)$$ (2)

where N represents the number of categories. If the cell category is the same as category C, the $y_{c}$ is 1; otherwise, it is 0. $\omega_{c}$ is the weight of category C. As the number of abnormal and normal cells are unbalanced (about 1:100∼1000) and we mainly focused on the abnormal cells, we oversampled the positive cases manually and used higher weights for two under-represented classes in the loss function. $p_{c}$ represents the probability that the prediction cells belong to category C. $L_{reg}\left(t_{i},t_{i}^{*}\right)$ is the robust smooth-*L*1 function which is defined as:

$${Smooth}_{L1}\left(x\right)=\left\{\begin{array}{l} 0.5x^{2},\left|x\right|<1\\ \left|x\right|-0.5,otherwise \end{array}\right.$$ (3)

where $t_{i}^{*}$ indicates that regression loss is only activated when the value of positive anchor ( $t_{i}^{*}$ ) is equal to unity. Therefore, the regression was used to calculate and minimize the deviation between the predicted target coordinates and ground-truth. The loss function of the mask branch (*L*_mask_) is defined as the average binary cross-entropy loss. The total loss function is the sum of all three branches and is defined as follows:

$$L=L_{cls}+L_{reg}+L_{mask}$$ (4)

This study implemented the Mask R-CNN workflow using TensorFlow 1.13.1 and the Keras 2.1.6 framework. ResNet50 was used as the backbone network. The coco pre-trained weights from the ImageNet dataset were used as the initial weights. The hyperparameters of the nucleus project in the matterport/Mask_RCNN GitHub repository were adopted as the starting point. For hyperparameter tuning, we checked the performance of models with different BACKBONEs, TRAIN_ROIS_PER_IMAGE, and MAX_GT_INSTANCES values in a small testing dataset and selected values that provided the best results. The 1000 manually labeled cervical cell images were divided into two groups (900 and 100) for training and test/validation. The Mask R-CNN model was trained using the training data set with the following parameters: epoch = 300, learning rate = 0.001.

### 2.6 Active learning method

To implement the active learning strategy, the data set was separated as follows. 1) First, 50 labeled images were randomly selected as the testing set for final validation. 2) Next, 100 manually labeled cervical cell images were randomly selected to train model A1 based on the initial ImageNet model in the first iteration. 3) Then, 200 images were randomly selected from the remaining 350 images and labeled automatically by model A1. These 200 labeled images were then revised by pathologists and used to train model A2 based on model A1 in the second round. 4) The remaining 150 images were annotated automatically by model A2 and were revised again by the pathologists to train model A3. The detailed processes of the model construction are shown in Figure 1B. Regarding the conventional labeling method, all 500 images were labeled manually, in which 450 images were selected randomly for model training and the remaining 50 images were used for validation. We compared the time and accuracy of data labeling and model prediction between conventional and active learning methods.

### 2.7 Patient classification models

The whole-slide images of 400 patients (∼3000 images per patient) with clinical diagnosis results were collected. These samples included 211 positive cases (187 atypical squamous cells of undetermined significance [ASCUS], 15 low-grade squamous intraepithelial lesions [LSILs], and 9 high-grade squamous intraepithelial lesions [HSILs]) and 189 negative cases (normal patients). These images were processed by the cell classification models to generate a cell number matrix of patient vs. cell types as training data for patient classification using the numbers of three types of cells as dependent variables and the clinical diagnosis results as the outcome to train the models. Four machine learning methods—logistic regression, random forest, SVM, and XGboost—were used to build the patient classification models and 10-fold cross-validation was applied for validation. The area under the curve (AUC) of ROC curves was used to compare the accuracy of these models and select the best model.

### 2.8 Model evaluation methods

A QC matrix adapted from the PASCAL VOC Challenge was used to compare the model performance (Everingham et al., 2010). The COCO Object detection challenge (Lin et al., 2014), which includes sensitivity (recall), specificity (precision), accuracy, positive predictive value (PPV), and negative predictive value (NPV), was defined as follows:

$$Specificity\;\left(precision\right)=\frac{TN}{TN+FP}$$ (5)

$$Sensitivity\;\left(recall\right)=\frac{TP}{TP+FN}$$ (6)

$$Accuracy=\frac{TP+TN}{TP+TN+FP+FN}$$ (7)

$$PPV=\frac{TP}{TP+FP}\;$$ (8)

$$NPV=\frac{TN}{FN+TN}$$ (9)

where TP, TN, FP, and FN indicate the numbers of true-positive, true-negative, false-positive, and false-negative cells or patients, respectively.

We also calculated the F1 scores, G-Mean, mean average precision (mAP), and mean average recall (mAR) to evaluate the object detection performance of all Mask R-CNN models. These values were calculated as follows:

$$F1=2\times \frac{Precision\times Recall}{Precision+Recall}$$ (10)

$$G-Mean=\sqrt{Recall\times Specificity}$$ (11)

$$mAP=\frac{1}{n}\overset{k=n}{\underset{1}{\sum }}AP_{k}$$ (12)

$$mAR=\frac{1}{n}\overset{k=n}{\underset{1}{\sum }}AR_{k}$$ (13)

In Eqs. 12, 13, the AP_k_ and AR_k_ represent the average precision and recall, respectively, of class k; n is the number of classes.

We used the AUC of the PR curve instead of the ROC curve to evaluate the accuracy and reliability of the cell classification model because PR curves are more appropriate for imbalanced datasets. The two axes of the PR curve refer to recall and precision. The curves are plotted according to the change in the probability threshold of the correct nuclear classification. AUC was used to measure the accuracy of cell classification. The formula for the AUC of the PR curves was as follows:

$$AUC=\underset{n}{\sum }\left(R_{n}-R_{n-1}\right)P_{n}$$ (14)

Here, R represents recall, P represents precision, and n represents an interval of probability threshold change. Additionally, we used the AUC scores of the ROC curves to summarize the performance of patient classification models.

### 2.9 Experimental environment

These experiments were performed on a Windows 10 PC with the following settings: CPU: third-generation core i5-3470@3.20GHz quad-core. Memory: 16 GB. Graphics card: NVIDIA GeForce GTX 1080 Ti 11 GB. Hard disk: 120 g SSD 1t HDD. The original Mask R-CNN deep learning framework (https://github.com/matterport/Mask_RCNN) was adapted to implement the cell segmentation and classification models. The software environment and libraries used in the project included anaconda 3, python 3.6, cuda 10.0, cudnn 7.5.

## 3 Results

### 3.1 Cervical cell segmentation and classification

**FIGURE 2:**
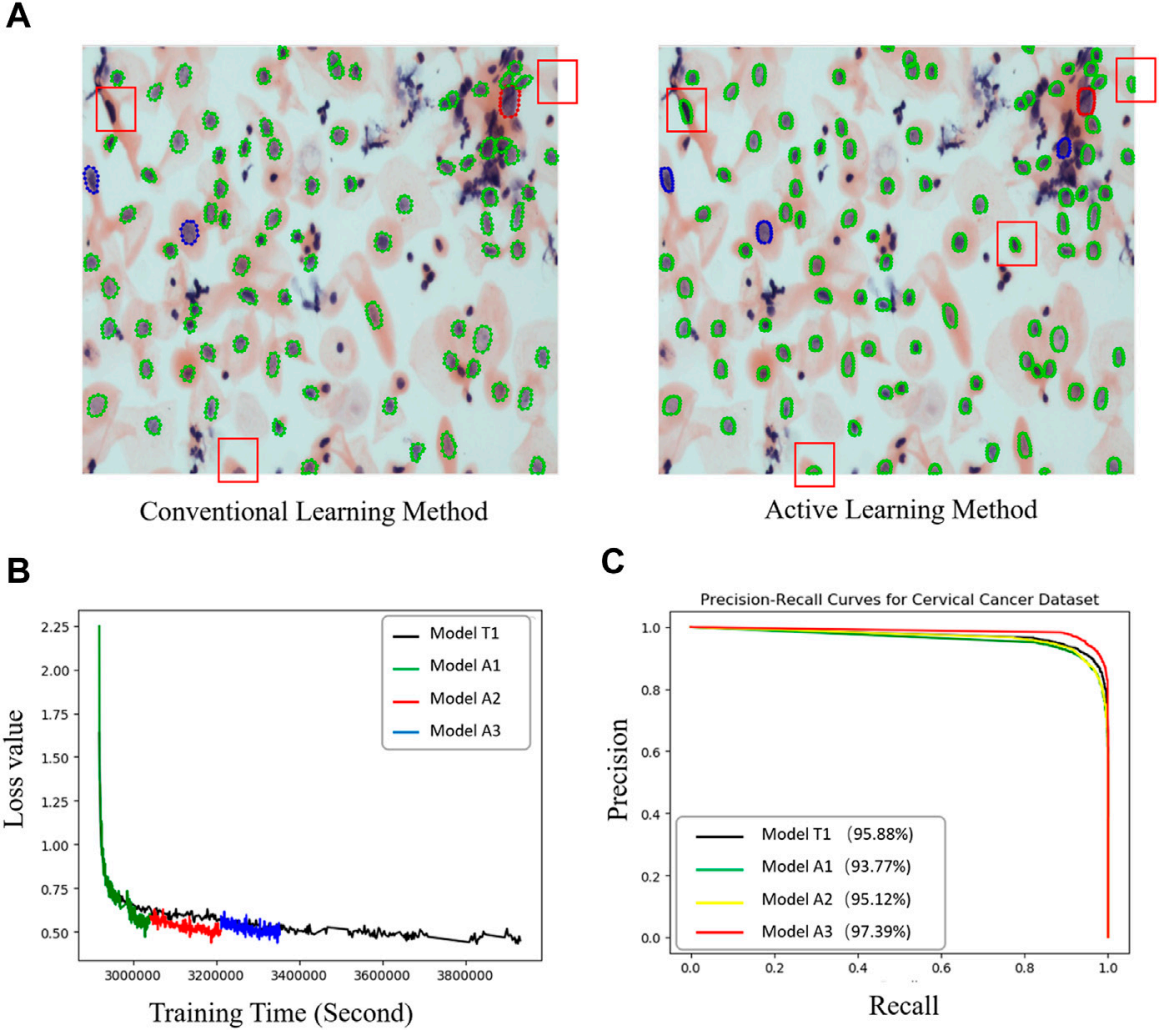
Performance comparisons between the active and conventional learning methods. **(A)** Comparison of nucleus segmentation results between manual annotation and active learning methods. Red and light blue boxes: nuclei missed in the manual annotation method. Orange boxes: nucleus with better contour segmentation by the active learning method compared to the traditional manual labeling method. **(B)** Loss curves of two methods (model T1: conventional method; A1, A2, and A3: active learning method) showing the faster regression for the active method. **(C)** PR curves of all models for cervical cell image recognition. The percentages in the legend are the prediction accuracies of the models.

We compared the performance of the conventional (manual labeling) learning method to the active learning method in terms of labeling time and accuracy. The results indicated that the active learning method could identify about 14% more cells and generated nucleus contours that better matched the nucleus boundaries compared to the conventional method (Table 1; Figure 2A). The active learning labeling methods showed an approximately four-fold acceleration in image labeling by performing certain repetitive tasks (such as contour labeling) by computer instead of human (Table 1). We also compared the performance of the two methods in model training. The results showed that the loss function of the active learning method converged faster than that of the traditional learning method (Figure 2B). Additionally, comparing the AUCs of the PR curves of the models showed that the overall effectiveness (precision and recall) of the active learning models (A1, A2, A3) improved gradually during the training iterations (Table 2). Intriguingly, the AUC, mAP, and F1 scores of model A3 were better than those of the T1 model, suggesting the advantages of the active learning method over the conventional learning method in prediction performance (Table 2). The results also showed that both T1 and A3 models achieved around 98% accuracy for cell classification in the test data. For normal (yin) cells, the sensitivity and specificity of the T1 and A3 model were approximately 97% and 66%, respectively, suggesting that some normal cells were mislabeled as abnormal cells. For abnormal cells, both T1 and A3 models achieved around 98% precision and recall (Tables 3, 4, 5). Taken together, these results demonstrate the advantages of the active learning strategy over the conventional supervised learning method in many perspectives, including image labeling speed and quality, the model training speed, and the prediction performance of the final model.

**TABLE 1:** Training and testing datasets.

Model	No. of training set	No. of testing set	Labeling time (h)	No. of cells (thousands)	No. of training epochs	Training time (h)
Model T1	450	50	75	19	300	282
Model A1	100	50	17		200	34
Model A2	200	50	1.3	23	150	47.5
Model A3	150	50	1		150	39.5

**TABLE 2:** Segmentation performance of four Mask R-CNN models (IoU threshold = 0.5).

Model	AUC (PR)	mAP (%)	mAR (%)	mF1 score
Model T1	95.88 ± 0.64	94.23 ± 0.25	95.11 ± 0.13	97.0
Model A1	93.77 ± 0.38	90.28 ± 0.21	81.02 ± 0.48	93.2
Model A2	95.12 ± 0.57	96.28 ± 0.50	95.29 ± 0.45	98.1
Model A3	97.39 ± 0.05	97.43 ± 0.62	94.36 ± 0.36	98.7

**TABLE 3:** The confusion matrix for cell classification generated by the T1 Model on the test dataset.

T1 Model		Predicted results			
		Yin	Yin-yang	Yang	Missing
Ground truth	yin	2839	36	17	23
	yin-yang	0	27	15	1
	yang	1	4	100	3

**TABLE 4:** Confusion matrix of cell classification of A3 Model in the test dataset.

A3 Model		Predicted results			
		Yin	Yin-yang	Yang	Missing
Ground truth	yin	2851	44	18	2
	yin-yang	0	28	15	0
	yang	1	0	106	0

**TABLE 5:** Cell classification performance in the test dataset.

Indicator	A3 Model			T1 Model
	Yin	Yang + yin-yang	Yin (%)	Yang + yin-yang
Sensitivity	97.80	99.33%	97.39	96.69%
Specificity	69.95	97.87%	66.37	98.18%
Accuracy	97.88	97.94%	97.49	98.11%

### 3.2 Patient classification

We applied the T1 and A3 models to classify all cells in the cervical cell smear WSIs of the 400 patients with final clinical diagnosis results. The numbers of the three types of cells and an additional four features (including patient age, the percentage of diseased cells, the sum of the numbers of suspected and diseased cells, and the percentage of suspected and diseased cells to the total cell count) were used as predictive features to train the patient classification models (Table 6). The clinical diagnosis results of patients were divided into two classes (normal and positive) and used as the outcomes.

**TABLE 6:** Features used for training the patient diagnosis models and their importance.

Feature name	Description	Importance (Model T1 + XGBoost)	Importance (Model A3 + XGBoost)
age	Patient age (years)	0.034	0.046
yin	Number of normal cells	0.017	0.031
yin-yang	Number of suspected diseased cells	0.047	0.030
yang	Number of diseased cells	**0.515**	**0.224**
yang_ratio	Percentage of diseased cells (number of diseased cells/total cell number)	**0.321**	**0.514**
combined_yang	Sum of the numbers of suspected and diseased cells	0.046	0.109
combined_yang_ratio	Percentage of suspected and diseased cells	0.018	0.043

Notice: The bold values are top features used by the patient diagnosis models.

We used four machine learning methods (logistic regression, random forest, SVM, and XGBoost) to construct the patient classification models. Then, 10x cross-validation was applied to evaluate the performance of the models. As Table 6 shows, the most important features were the number and percentage of diseased cells. More importantly, the validation results showed that all patient classification models based on cell classification results from model A1 showed better AUCs, accuracies, specificities, and sensitivities (except for SVM) than those based on cell classification data from model T1 (Table 7). These findings further strengthened our previous conclusion that the active learning model can provide higher-quality cell classification results compared to the conventional supervised learning method (Table 7).

**TABLE 7:** Performance comparison of four machine learning algorithms.

Patient classification algorithm	Model	AUC (%)	Accuracy (%)	Specificity (%)	Sensitivity (%)	PPV/NPV (%)	F1 score	G-Mean
Logistic regression	Model T1	96.28 ± 2.6	90.0 ± 4.1	97.89 ± 3.5	82.96 ± 8.2	97.9/84.2	89.56 ± 4.6	89.9 ± 4.2
	Model A3	98.87 ± 0.8	93.7 ± 4.7	98.47 ± 1.5	88.61 ± 9.3	99.5/89.3	93.45 ± 5.4	93.7 ± 5.0
SVM	Model T1	96.66 ± 2.6	83.7 ± 10.8	79.47 ± 28.2	87.66 ± 12.8	87.3/88.9	85.57 ± 8.3	80.8 + 15
	Model A3	99.14 ± 0.6	89.2 ± 7.7	96.31 ± 9.4	83.09 ± 15.4	97.3/85.6	88.44 ± 9.3	88.7 ± 8.4
Random forest	Model T1	95.0 ± 4.29	91.0 ± 4.8	93.16 ± 5.2	89.09 ± 7.4	93.6/88.9	91.14 ± 5.0	90.9 ± 4.9
	Model A3	99.48 ± 1.0	97.0 ± 2.2	98.94 ± 2.1	95.26 ± 4.2	99.0/95.1	97.05 ± 2.2	97.0 ± 2.1
XGBoost	Model T1	96.60 ± 2.9	92.5 ± 4.2	93.16 ± 6.2	91.92 ± 5.2	93.9/91.4	92.79 ± 4.0	92.4 ± 4.3
	Model A3	99.45 ± 0.6	97.5 ± 2.2	98.95 ± 2.1	96.21 ± 4.1	99.0/96.0	97.56 ± 2.2	97.5 ± 2.2

We then compared the performance of four patient classification methods by performing Friedman tests using the accuracy, specificity, sensitivity, AUC, and F1 cores of the10x cross-validation data, which showed significant differences in accuracy, sensitivity, AUC, and F1 when using cell classification results from the A3 model. The accuracy, specificity, sensitivity, and F1 score also differed significantly when using cell classification results from the T1 model (Supplementary Table S1). Table 7 shows that the random forest and XGBoost models have better AUCs, accuracies, specificities, and sensitivities than the logistic regression and SVM models. The random forest and XGBoost models showed comparable performances. Intriguingly, the AUCs, accuracies, specificities, and sensitivities of both random forest and XGBoost models were above 99.4%, 97.0%, 98.9%, and 95.2% respectively, suggesting that both models achieved good performance (Table 7, Supplementary Table S2, S3, and Supplementary Figure S1). We also used Wilcoxon tests to compare the performance of the different methods. The results showed that the XGBoost and random forest models outperformed the SVM and logistic models in terms of accuracy and other metrics. (Supplementary Table S4).

## 4 Discussion

This study preliminarily explored the feasibility of an active learning strategy in the construction of deep learning models for pathological images. Moreover, we proposed and utilized an active learning strategy to improve the speed and quality of pathological image annotation of CTC, further comparing the accuracy and efficiency between this strategy and a conventional supervised learning strategy in the construction of a deep learning model. The findings of this study demonstrated that the active learning strategy has significant advantages over conventional supervised learning in the quality and speed of image annotation, the training speed of the model, and the accuracy of the final model, which can be applied widely in the construction of other pathological diagnosis models.

Compared to previous works, which usually focused on either cell classification or patient classification problems, our method solved both using Mask R-CNN combined with XGBoost and achieved higher or comparable accuracy, sensitivity, and specificity (Supplementary Table S5) (Wu et al., 2018; Sompawong et al., 2019; Tan et al., 2021; Wentzensen et al., 2021). Moreover, our model achieved high sensitivity (96.2%) and specificity (98.95%) in the prediction of cervical cancer patients, which are absolutely required to avoid missing any cancer patients and reduce the follow-up cost of false-positive cases.

However, our proposed model also has several limitations. First, although DCCNs are better than traditional algorithms at cell segregation, it was still difficult to differentiate diseased and normal cells from overlapping and adhesion cells; hence, the accuracy was relatively low, especially when the quality of images was low. Second, the collected dataset lacked sufficient samples to train a model to predict pathological subtypes such as LSIL and HSIL. Therefore, future research should extend this study by collecting more training data from different hospitals to increase the data diversity and improve the model’s robustness. The active learning strategy may be helpful to accelerate the labeling of larger data. The cell classification/labeling system must also be extended to more specific cell types such as squamous metaplastic cells and endocervical cells. Our models may also be used to predict the cytology grades of patient lesions (such as ASCUS, LSIL, and HSIL). Moreover, considering more relevant patient information, such as patient age, HPV infection status, vaginal inflammation status, and lifestyle or environmental factors may help to further improve the model accuracy. More importantly, validating these models in prospective clinical studies is urgently needed to ensure that the models can be generalized to real-world clinic data.

## 5 Conclusion

In conclusion, the current study presents a DCCN model that showed high accuracy, sensitivity, and specificity in cervical cancer screening and diagnosis using TCT images. The active learning strategy showed greater advantages over conventional supervised learning in reducing the cost of image labeling and improving the training dataset quality and model accuracy. This strategy could be easily applied to the construction of AI diagnosis models of other diseases.

## Data Availability

The datasets presented in this study can be found in online repositories. The names of the repository/repositories and accession number(s) can be found below: https://github.com/hqyone/cancer_rcnn.
