# Supplementary material for: Deep convolutional neural networks using an active learning strategy for cervical cancer screening and diagnosis
Source: Front Bioinform. 2023 Mar 9;3:1101667. doi: 10.3389/fbinf.2023.1101667 (PMC10034408; doi:10.3389/fbinf.2023.1101667)
Supplement: Supplementary file 1 [file Presentation1.pdf]

Supplemental Materials

Deep Convolutional Neural Networks Using Active Learning Strategy for Cervical Cancer Screening and Diagnosis

Xue-guang Li, Ming-yue Du, Shan-ru Zhou, Ming-qing Zhou, Qi-yao Peng, Zi-yao Chen, Jun-hua Zhou*, Quanyuan He*

Table S1. Confusion matrix of diagnosis results of T1 combing XGBoost model vs pathologist in test samples

	**Model T1+XGBoost**		
**Pathologist**	Positive	Negative	Total
Positive	N=34	N=4	N=38
Negative	N=2	N=40	N=42
Total	N=36	N=44	N=80

Table S2. Confusion matrix of diagnosis results of A3 combing XGBoost model vs pathologists in test samples

	**Model A3+XGBoost**		
**Pathologist**	Positive	Negative	Total
Positive	N=38	N=0	N=38
Negative	N=1	N=41	N=42
Total	N=39	N=41	N=80

Table S3. Test the performance difference of four patient classification models using Friedman test.

Cell classification Model	Accuracy	Specificity	Sensitivity	AUC	F1
A3	**0.000107**	0.732528	**0.000215**	**0.030374**	**0.000107**
T1	**0.017989**	**0.013808**	**0.007021**	0.33626	**0.014649**

Table S4. Statistical comparison of the performance measurement between different ML models using Wilcoxon test.

Cell classification Model	Performance Measurement	XGBoost vs SVM	XGBoost vs Logistic reg	Random Forest vs SVM	Random Forest vs Logistic reg	XGBoost vs Random Forest	SVM vs Logistic reg
A3	Accuracy	**0.0058**	**0.0058**	**0.0057**	**0.0079**	0.1586	0.9642
	Specificity	0.327	**0.0053**	0.3273	0.841	1.0	0.8413
	Sensitivity	**0.0089**	0.8413	**0.0088**	**0.0087**	0.1586	0.9346
	AUC	**0.0462**	**0.0057**	0.1700	0.0615	0.8816	0.1465
	F1	**0.0053**	**0.0178**	**0.0058**	**0.0089**	0.1586	0.9771
T1	Accuracy	**0.0073**	**0.0582**	**0.010**	0.278	**0.0417**	0.9673
	Specificity	0.123	0.9872	0.154	0.991	1.0	0.9789
	Sensitivity	0.220	**0.0037**	0.362	**0.0086**	**0.021557**	0.1548
	AUC	0.570	0.5236	0.975	0.919	**0.025306**	0.1875
	F1	**0.0054**	**0.032**	**0.008**	**0.0087**	**0.023200**	0.9306

Table S5. Comparisons between our study with others in terms of data, methods, and the performance of cell classification and patient classification

Reference	Training and test data	Methods	Cell classification	Patient classification:
**Wentzensen et al., 2021 [1]**	238 slides/2671 images	CNN4/IncV3	NA	Sen:91.3%<br>Spec:46.1%
**Tan et al., 2021 [2]**	290 slides/424106 image	Faster R-CNN	NA	Sen: 99.4%,<br>Spec :34.8%
**Sompawong et al., 2019 [3]**	178 images	Mask R-CNN	Acc: 89.8%<br>Sen: 72.5%<br>Spec:94.3%	NA
**This Study**	400 slides/1000 images	Mask R-CNN + XGBoost	For positive cells<br>Acc: 97.9%<br>Sen: 99.3%<br>Spec: 97.9%	Sen: 96.2%,<br>Spec:98.95%

1. Wentzensen, N. *et al.* Accuracy and Efficiency of Deep-Learning-Based Automation of Dual

Stain Cytology in Cervical Cancer Screening. *J. Natl. Cancer Inst.* **113**, 72–79 (2021).

2. Tan,X., Li,K., Zhang,J., Wang,W., Wu,B., Wu,J., Li,X. and Huang,X. (2021) Automatic model for cervical cancer screening based on convolutional neural network: a retrospective, multicohort, multicenter study. *Cancer Cell Int.*, **21**, 1–10.
3. Sompawong,N., Mopan,J., Pooprasert,P., Himakhun,W., Suwannarurk,K., Ngamvirojcharoen,J., Vachiramon,T. and Tantibundhit,C. (2019) Sci-Hub | Automated Pap Smear Cervical Cancer Screening Using Deep Learning. 2019 41st Annual International Conference of the IEEE Engineering in Medicine and Biology Society (EMBC) | 10.1109/embc.2019.8856369. *2019 41st Annu. Int. Conf. IEEE Eng. Med. Biol. Soc.*

Figure S1：ROC and PR curves of T1/ A3 model combining with random forest model to identify cervical cancer patients.

Model T1 + Random Forest：

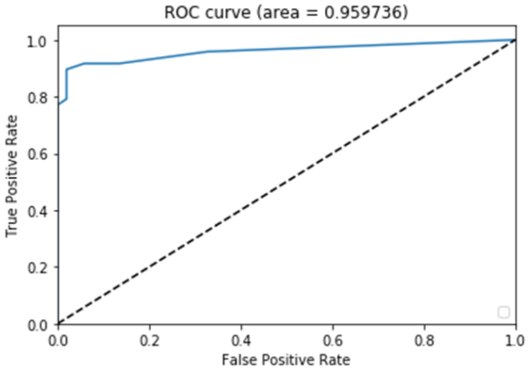

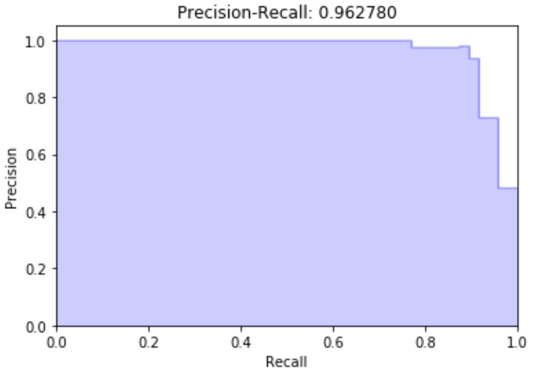


```
Confusion matrix:
 [[136   1]
 [  2 161]]
Training AUC: 99.9798 %
Training accuracy: 99.0000 %
Test Confusion matrix:
 [[51  1]
 [ 6 42]]
Test Training AUC: 95.9736 %
Test Training accuracy: 93.0000 %
Average_precision: 0.9628
```

Model A3 + Random Forest：

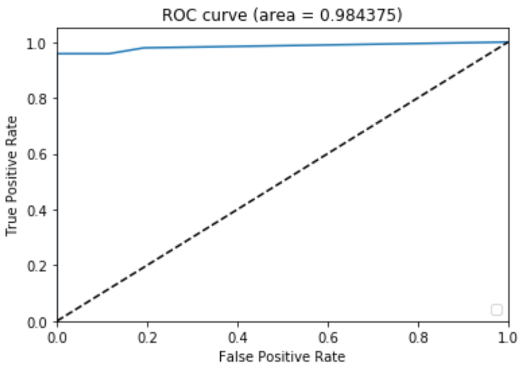

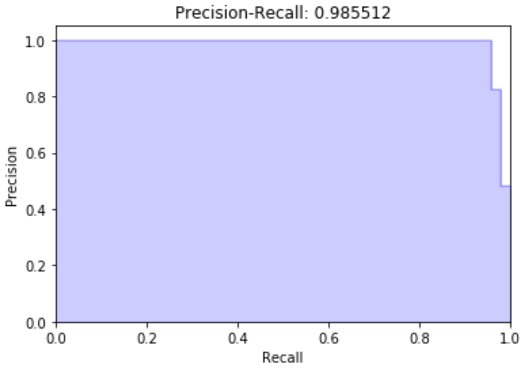

```
Confusion matrix:
 [[137   0]
 [  2 161]]
Training AUC: 100.0000 %
Training accuracy: 99.3333 %
Test Confusion matrix:
 [[50  2]
 [ 2 46]]
Test Training AUC: 98.4375 %
Test Training accuracy: 96.0000 %
Average_precision: 0.9855
```

Figure S2: ROC and PR curves of the T1/ A3 models combining with logistic regression to identify cervical cancer patients.

Model T1 + logistic regression

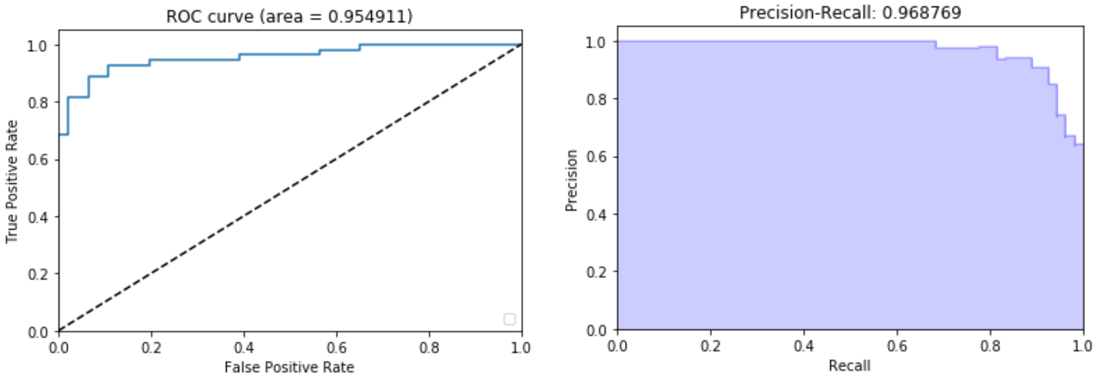


```
Confusion matrix:
 [[143   0]
 [ 28 129]]
Training AUC: 97.0469 %
Training accuracy: 90.6667 %
Test Confusion matrix:
 [[43  3]
 [ 9 45]]
Test Training AUC: 95.4911 %
Test Training accuracy: 88.0000 %
Average_precision: 0.9688
```

Model A3 + logistic regression

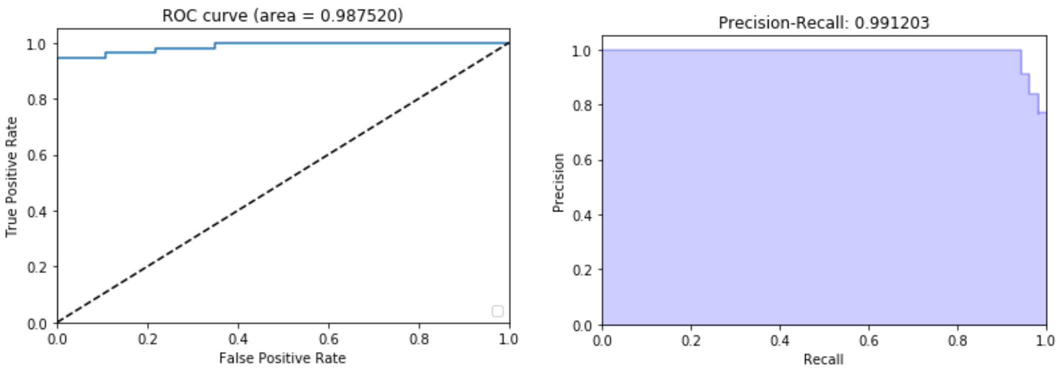

```
Confusion matrix:
 [[143   0]
 [ 20 137]]
Training AUC: 99.2161 %
Training accuracy: 93.3333 %
Test Confusion matrix:
 [[46  0]
 [ 8 46]]
Test Training AUC: 98.7520 %
Test Training accuracy: 92.0000 %
Average_precision: 0.9912
```

Figure S3: ROC and PR curves of the T1/ A3 models combining with SVM to identify cervical cancer patients.

Model T1 + SVM model

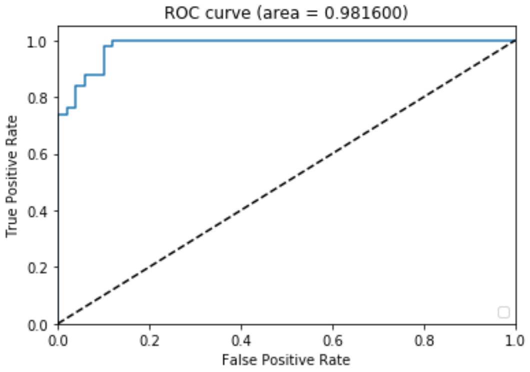

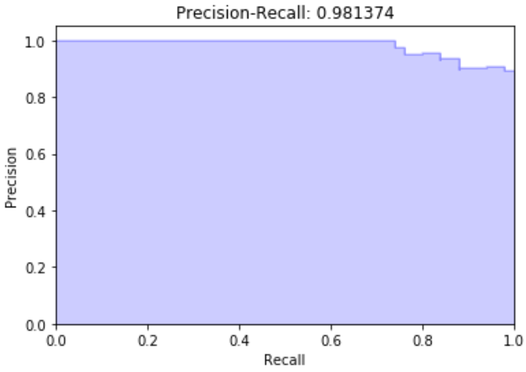


```
Confusion matrix:
 [[137   2]
 [ 42 119]]
Training AUC: 96.1035 %
Training accuracy: 85.3333 %
Test Confusion matrix:
 [[50  0]
 [13 37]]
Test Training AUC: 98.1600 %
Test Training accuracy: 87.0000 %
Average_precision: 0.9814
```

Model A3:

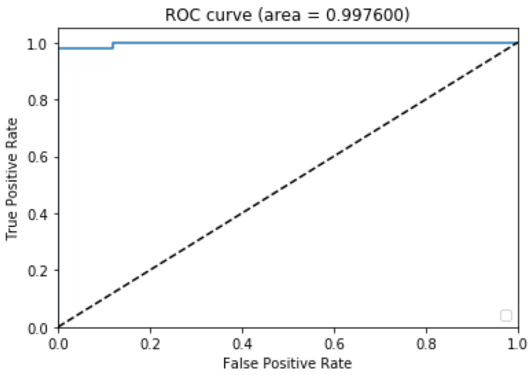

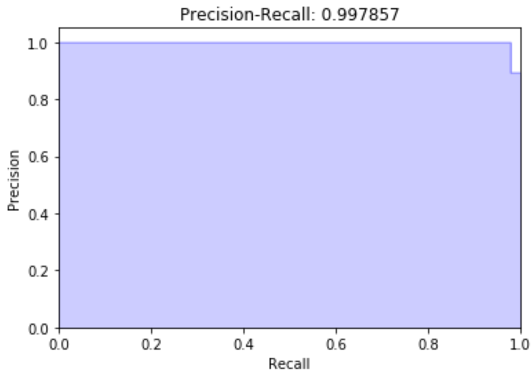

```
Confusion matrix:
 [[122  17]
 [  3 158]]
Training AUC: 99.4772 %
Training accuracy: 93.3333 %
Test Confusion matrix:
 [[45  5]
 [ 1 49]]
Test Training AUC: 99.7600 %
Test Training accuracy: 94.0000 %
Average_precision: 0.9979
```

Figure. S4. ROC and PR curves of the T1/ A3 models combining with XGBoost to identify cervical cancer patients

Model T1 + XGBoost:

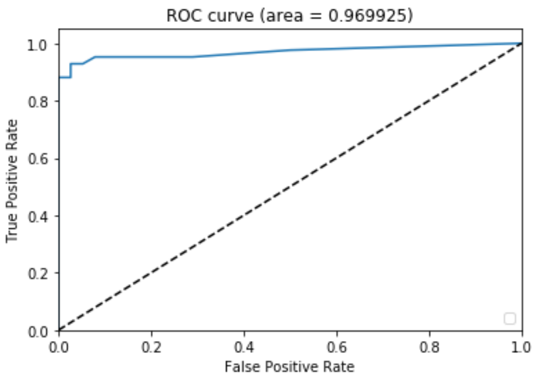

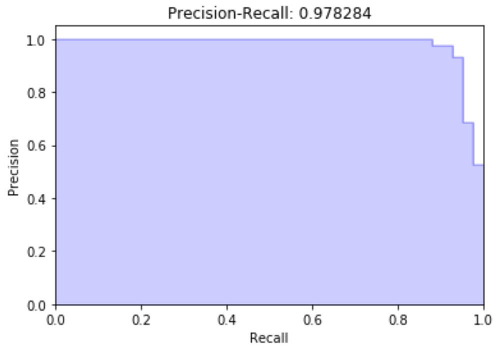


```
Confusion matrix:
 [[142   9]
 [ 10 159]]
Training AUC: 96.9670 %
Training accuracy: 94.0625 %
Test Confusion matrix:
 [[34  4]
 [ 2 40]]
Test Training AUC: 96.9925 %
Test Training accuracy: 92.5000 %
Average_precision: 0.9783
```

Model A3 + XGBoost:

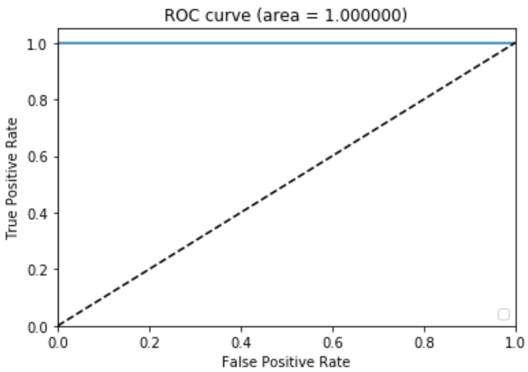

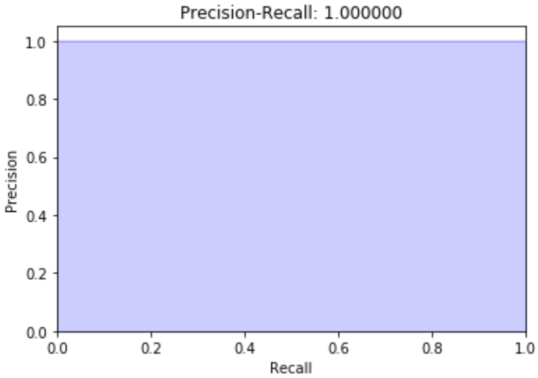

```
Confusion matrix:
 [[150   1]
 [ 11 158]]
Training AUC: 99.8981 %
Training accuracy: 96.2500 %
Test Confusion matrix:
 [[38  0]
 [ 1 41]]
Test Training AUC: 100.0000 %
Test Training accuracy: 98.7500 %
Average_precision: 1.0000
```
